# Assessing Emotional Responses to the Spatial Quality of Urban Green Spaces through Self-Report and Face Recognition Measures

**DOI:** 10.3390/ijerph18168526

**Published:** 2021-08-12

**Authors:** Lin Qiao, Jingwei Zhuang, Xuan Zhang, Yang Su, Yiping Xia

**Affiliations:** 1 Institute of Landscape Architecture, College of Agriculture and Biotechnology, Zhejiang University, Hangzhou 310058, China; lynnq@zju.edu.cn (L.Q.); 21916209@zju.edu.cn (J.Z.); zhang_xuan@zju.edu.cn (X.Z.); 2 Institute of Urban and Rural Planning Theories and Technologies, College of Civil Engineering and Architecture, Zhejiang University, Hangzhou 310058, China; 3 The Architectural Design & Research Institute of Zhejiang University Co, Ltd., Hangzhou 310030, China; suyang@zju.edu.cn

**Keywords:** urban green space, spatial quality, emotional responses, structural attributes, face recognition

## Abstract

Although creating a high-quality urban green space (UGS) is of considerable importance in public health, few studies have used individuals’ emotions to evaluate the UGS quality. This study aims to conduct a multidimensional emotional assessment method of UGS from the perspective of spatial quality. Panoramic videos of 15 scenes in the West Lake Scenic Area were displayed to 34 participants. For each scene, 12 attributes regarding spatial quality were quantified, including perceived plant attributes, spatial structure attributes, and experiences of UGS. Then, the Self-Assessment-Manikin (SAM) scale and face recognition model were used to measure people’s valence-arousal emotion values. Among all the predictors, the percentages of water and plants were the most predictive indicators of emotional responses measured by SAM scale, while the interpretation rate of the model measured by face recognition was insufficiently high. Concerning gender differences, women experienced a significantly higher valence than men. Higher percentages of water and plants, larger sizes, approximate shape index, and lower canopy densities were often related to positive emotions. Hence, designers must consider all structural attributes of green spaces, as well as enrich visual perception and provide various activities while creating a UGS. In addition, we suggest combining both physiological and psychological methods to assess emotional responses in future studies. Because the face recognition model can provide objective measurement of emotional responses, and the self-report questionnaire is much easier to administer and can be used as a supplement.

## 1. Introduction

The world is experiencing progressive urbanization. Cities continue to expand, and residents are increasingly moving away from natural environments. A high quality Urban Green Space (UGS) is indispensable for improving the urban environment. An increasing number of research findings have pointed to UGSs as a resource for promoting public health. It is commonly believed that contact with nature may enhance positive emotions, restore attention, improve cognitive ability and reduce stress and anxiety [1,2,3,4,5]. UGSs provide relatively low-cost opportunities for residents to connect with nature in their daily lives. During the COVID-19 pandemic, the latest research has found that what many people missed most was close contact with nature, such as exercising outdoors and meeting other people [6,7,8,9]. In other words, UGSs are seen as vital places.

As mentioned in environmental psychology studies, human beings’ mental health can be affected by the surrounding environment [10,11]. Despite the growing recognition of the health benefits of UGSs, the previous studies highlighted a lack of evidence investigating the role of UGS quality rather than provision. It is likely due to the difficultly in identifying the terms of UGS quality, and the time and cost of measuring quality across all UGSs within a study area in a comprehensive and systematic approach [11]. Definitions of UGS quality differ but broadly deal with the various attributes that create a green space, and the activities they support. Annerstedt et al. (2012) identified the following qualities of green spaces: serene, wild, lush, spacious, or culture [12]. It is also possible to use the ecological, microclimatic, and social purposes to assess urban green spaces benefits from the multidimensional perspective [13]. And many studies concurred that landscape features, facilities, amenities, and maintenance are indicative of a green space quality [14]. In this paper, we mainly focus on the spatial quality of UGS.

The quantitative assessment of the spatial quality of UGS requires the use of secondary indicators. Researchers have variously suggested relationships between individual health and spatial quality characteristics of UGS, such as size [15], location [16,17], space type [18,19], vegetation cover [20], or Normalized Difference Vegetation Index (NDVI) [21]. Also, it could be that the mental health benefits of green spaces are influenced by how these green spaces are perceived. Seasonal color [22,23], plant arrangement [23] and perceived naturalness [24,25] also played important roles in the spatial quality of UGSs. Furthermore, evidence from previous studies suggests that the physical and mental health benefits from UGSs are less a matter of their structural attributes than their nonstructural dimensions, i.e., individuals’ experiences when they visit UGSs [26,27]. This characteristic makes sense, as the domains of the spatial quality of UGSs should include both spatial characteristics and spatial experiences [14,28]. However, most of these studies have only focused on the presence of attributes in UGSs, overlooking the quantitative analysis of UGSs’ spatial quality. And some of the adopted attributes are coarse, indicating large aggregations in spatial features, which may be only suitable for large scale UGSs.

In addition, the current research on the spatial attributes of UGS is mainly focused on mental restoration and aesthetic preference, and few studies have used emotional dimensions to evaluate the spatial quality of UGS [17,29,30,31]. This omission may be due to the complexity of personal emotional expression and the difficulty of capturing emotional responses. There is a need to explore the spatial quality of UGSs to identify which attributes of green spaces have the potential to promote individuals’ emotional well-being.

Given the importance of emotional responses in evaluating the quality of UGS, the measurement of emotion related to UGS is a crucial issue and is acknowledged as a complex research task. Selecting an appropriate evaluative dimension of emotion is also necessary to collect reliable and valid data. Most relevant studies regard emotion as a multidimensional structure and assess it through valence and arousal dimensions [32]. Valence refers to the pleasantness of an experience, and ‘pleasant’ and ‘unpleasant’ usually anchor the continuum of the valence dimension. Arousal, on the other hand, refers to the activation of the internal state and usually contrasts states of ‘quiet’ with states of ‘excited’. Dimensional frameworks appear to have substantial explanatory value and can better capture human beings’ emotional responses. Given this capacity, a dimensional approach to emotion serves as the appropriate theoretical basis for further discussion of the psychophysiological measurement of emotion in this study.

In accordance with past studies, emotional measurement techniques can be divided into two main categories: psychological (subjective, understanding participants by conducting self-report, interviews, etc.) and physiological (objective, counting and analyzing emotional responses statistically through biosensors, software algorithms, etc.) [33]. Self-report measures are simple and quick methods to collect individuals’ emotional reactions [34,35]. In these methods, respondents are asked to describe their emotional responses through open-ended questions or rate their emotional state on a set of affective items. The most popular dimensional approach is the Self-Assessment Manikin (SAM) [36]. In recent years, artificial intelligence (AI) tools have emerged and greatly contributed to data collection. Face recognition is a novel technique that analyzes visual recordings of faces through a software algorithm that was generated by training the model using big data of intended emotional expressions. The model performs even better than humans in face recognition benchmark testing because of the emergence of neural networks [37,38]. Current technology can achieve an accuracy of facial analysis in as high as 87% of the perceived emotion [16]. Svoray et al. (2018) collected photos of faces and analyzed the relationship between human facial expressions and exposure to nature [39]. Meng et al. (2019) explored the effectiveness of facial expression recognition for evaluating urban sound perception. However, emotion recognition remains a complex problem [35]. The use of neural networks allows us to exceed the accuracy of manual identification [40], but each additional percentage of accuracy is achieved through complex scientific research and experiments [41].

The spatial quality of UGS should incorporate comprehensive attributes, involving both physical elements and subjective components, to develop a truly quantitative and objective evaluation methodology. Hence, we investigate 3 types of spatial quality of UGSs, including spatial structure attributes, perceived plant attributes and experiences of UGS, which can affect people’s emotional responses. Our study uses physiological and psychological measures to identify relevant spatial qualities of UGS as the predictors of emotion assessment dimensions. Then, we attempt to assess the specific impact of quantitative structural attributes on emotional responses. This research analyzes gender differences in people’s emotions in UGSs. Finally, we explore the similarities and differences between self-report and face recognition methods. 

## 2. Materials and Methods

### 2.1. Site Selection

**Figure 1:**
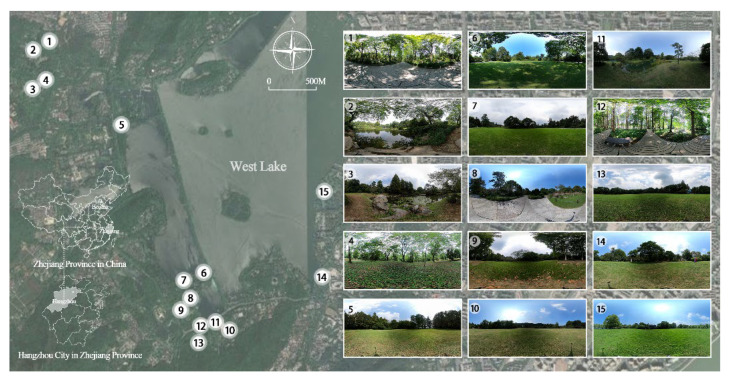
The locations and 360° panoramic photos of the 15 study sites in Hangzhou, China.

The study was conducted in Hangzhou, Zhejiang Province, East China (Figure 1). West Lake is a World Cultural Heritage famous for its beautiful and elegant lakes and mountains, as well as its rich cultural heritage. On the basis of the preliminary surveying and mapping of the West Lake scenic area, we chose cases for analysis primarily based on plant landscape construction. Moreover, the sample plots were selected according to the spatial distribution. The green spaces there have good representativeness and research value. We selected a specific type of UGS in the West Lake area that were composed of different attributes of spatial quality (Table A1). The majority of them were along the road or on the lawn edge, the others were part of natural spaces surrounded by plants. Some include water features, some have dense vegetation, and a few are located beside walking pathways, creating green spaces that can be used for a variety of activities. The site was treated as a random effect to control for independence [42]. A total of 15 sample plots were selected based on sampling selection.

### 2.2. Participants and Design

We recruited 34 participants through website forums. They were almost all college students, aged between 18 and 33 (18 females). The age range was between 18 and 33 years, with a mean of 24 years. The participants were healthy enough to complete the entire experiment. Participation in the research was voluntary, and volunteers were paid a certain amount. We performed a variance analysis within group to detect whether people’s valence and arousal of 15 scenes were consistent. The results showed that there were no significant differences within the groups. Shows that the sample selected for this study is reasonable. The study was performed with the approval of the local Management Committee of Hangzhou West Lake Scenic Area and Zhejiang University, China.

### 2.3. Environmental Simulation

**Figure A1:**
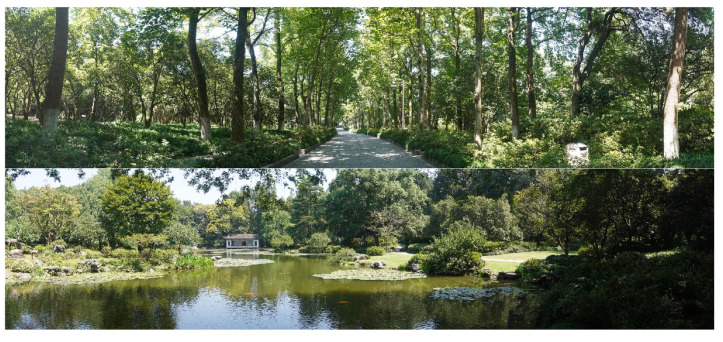

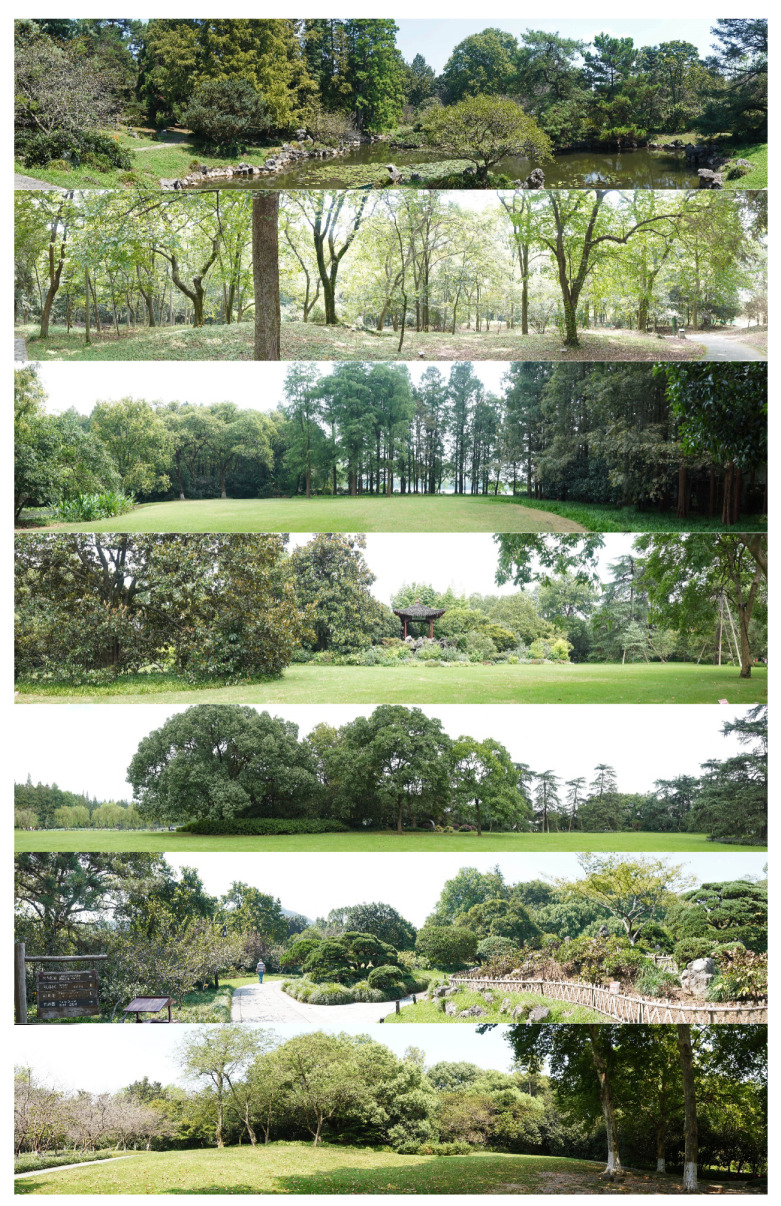

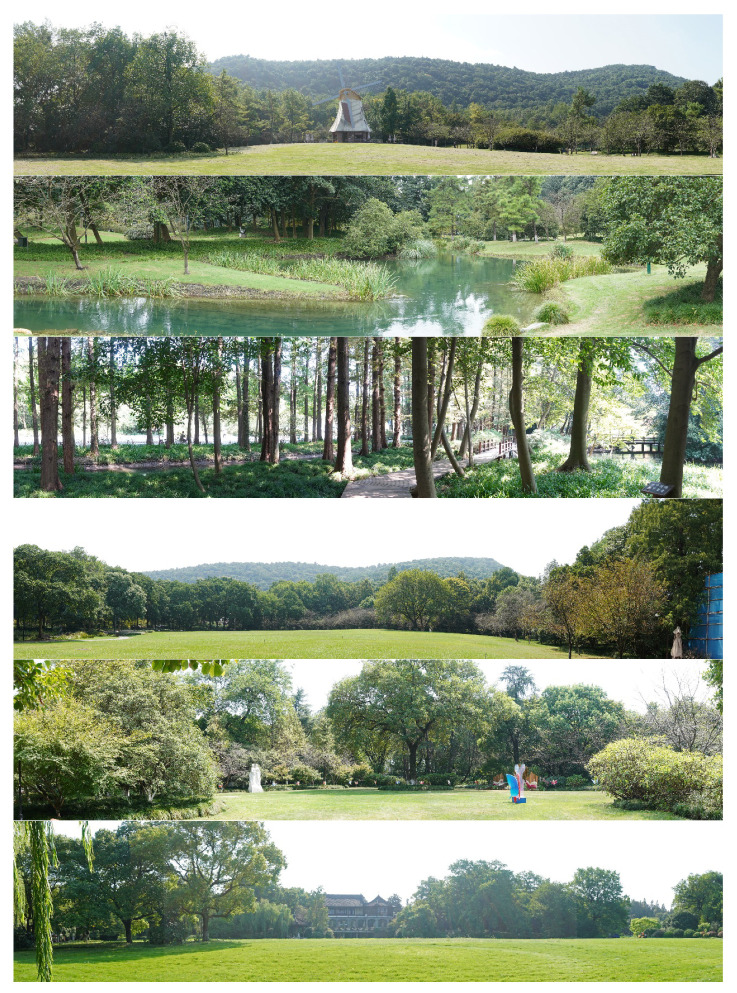
15 horizontal panoramic photos and the website address of 15 360° panoramic videos.

Scenic photos were taken on sunny days from August to September 2020, and the shooting time was between 9:00 and 16:00. The location of the photographer in the UGSs and the photographic views were selected randomly, but it was confirmed that the photographs could capture the principal characteristics of the scenes, such as vegetation type, color density, and architectural structure. Several photographs were taken at each sample site, but only one picture of good quality (e.g., a good photographic angle, clear contrast) representative of each site was selected by the authors for the experiment. The selected photos were preprocessed to improve their accuracy, including calibration of image brightness and chroma. GoPro MAX cameras were used to take 360° panoramic photos of each scene. The photos had an aspect ratio of 3:2. The GoPro was set up on a tripod at a height of approximately 62 cm to shoot. There were no large trees or other visual impairments within 10 m of the camera’s field of view, and the photos accurately showed the entire field of view captured by the camera. Then, the photos were imported into the VRyun website (http://www.vryun.work, accessed on 6 August 2021) to generate panoramic videos. Finally, a total of 15 one-minute video segments were produced. What’s more, SONY ILCEA 6400 camera was used to take the horizontal panoramic photos, with an aspect ratio of 8192 × 1856 pixels (Figure A1, Appendix A). We always kept the camera lens level when shooting and fixed it at a height of 160 cm from the ground. These photographs were used for subsequent analysis.

### 2.4. Measures

#### 2.4.1. Spatial Quality of UGS

**Figure A2:**
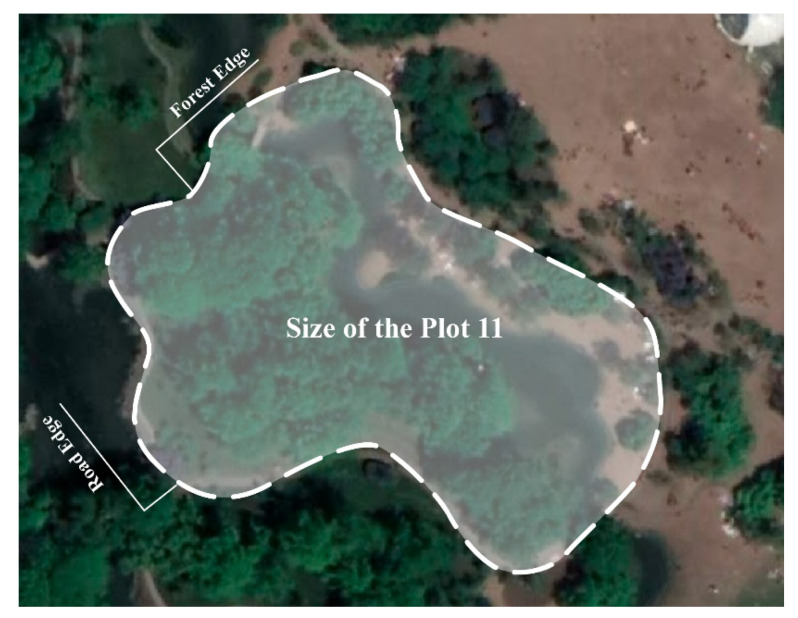
The schematic diagram of field measurement methods.

We proposed that measuring spatial variables based on the perspectives of the UGS users themselves is more meaningful. Considering previous similar studies, the spatial quality attributes were summarized at the outset via the literature review [20,43,44]. Spatial quality attributes that are specific to specific country and region cases, or where the size of green space is too large or too small, are excluded. For the selected attributes, a preliminary correlation analysis and regression analysis was performed, those that were unrelated to emotional responses or had multicollinearity were excluded. Finally, 5 spatial structure attributes (size, shape index of edges, canopy density, percentage of water, percentage of plants) and 5 perceived plant attributes (vegetation layers, shape of trees, texture of trees, color diversity and cultural connotation) of UGS were chosen. In addition, this study also includes the evaluation of 2 nonstructural attributes of UGS experiences (use function and visit frequency). The spatial structure attributes were mainly obtained through field measurement. We measured the length of each side and the canopy of trees in green spaces using a distance finder and a tape. The boundaries of the regular-shaped plots were defined by the inner edges of the road or the lawn, whereas the others (including plots 6, 7, 11, 13) were defined by the forest outermost edges (Figure A2). We then computed their area, perimeter, shape index of edge, and canopy density based on the data. The percentage of water and the percentage of plants were measured by the number of pixels in each of the horizontal panoramic photos [45,46]. Then, the dummy-coding method was employed to quantify 5 spatial structural attributes, referring to previous literature [29,30,43]. The evaluation criteria for the structural attributes of UGS are shown in Table 1. The perceived plant attributes were measured by a photo questionnaire. The participants were asked to evaluate the plant attributes of each plot on a Likert scale (1 = strongly disagree; 5 = strongly agree). The Cronbach’s α is 0.843. In response to the nonstructural quality posed after the presentation of the UGS scenes, we designed questions about the visit motivation of each scene. We asked, “Do you think any factor shown in the video will motivate you to use the scene?” Answers were in the form of binary variable data, with “1” representing “yes” and “0” representing “no”. Then, we evaluated the use function of the UGS by adding all 6 scores. Finally, we asked “How often do you visit UGSs?’’ (Questionnaire 1).

#### 2.4.2. Physiological Measures

For the collection of facial data, participants were required to sit in front of a well-lit window to allow better detection of facial movement. They were asked not to wear thick-rimmed glasses or anything that might cover their faces. Then, we captured the facial movements of the participants with camera videos. To recognize facial expressions of the video we captured, we used the face recognition model proposed by Do et al., 2020 [40]. After we input the videos to the model, the valence-arousal emotion values were output for the subsequent analysis. There were 3 steps to get the final results. The premise of emotion recognition was to detect faces, so the faces in the video frames were detected and aligned in the first step of the pre-processing process. The facial features were then extracted, and the facial expression categories and valence-arousal values were calculated by ResNet50 (a convolutional neural network which is 50 layers deep). Finally, the valence and arousal values were set to range from 0 to 100 and output in seconds. In our experiment, we also attempted to improve the model’s transferability and robustness by re-labeling the distinctly incorrect results of face emotion in our videos during the pre-processing step (cf. Section 2.5).

#### 2.4.3. Psychological Measures

After the experimental procedure, the participants were allowed to describe their feelings through questionnaires. Based on the dimension of emotion evaluation, a Self-Assessment-Manikin (SAM) was used to measure their emotional responses. The SAM is a nonverbal, pictorial assessment technique that directly measures valence and arousal in response to various environmental stimuli, which reduces the potential influence of laziness on the questionnaire results. Backs et al. (2005) proved that the various dimensions of SAM had high internal consistency [47]. Cronbach’s α coefficient of valence and arousal dimension were 0.98 and 0.63, which was widely used in the emotional evaluation research of adolescents, college students, and normal elderly in China [48,49]. The two related dimensions, valence and arousal, are represented by different human-shaped pictures. There are five human-shaped pictures in each dimension in the picture. The numbers in the table below represent different degrees of components from 1–9. From top to bottom, the first dimension is valence. The numbers 1, 3, 5, 7, and 9 represent frown (a frowning figure), unhappy, neutral, smiling, and happy (a smiling figure). The second dimension is arousal. The numbers 1, 3, 5, 7 and 9 indicate sleepy (eyes closed), drowsy, neutral, excited, and stimulation (eyes wide open). Dimensions 2, 4, 6, and 8 provide volunteers with subtler choices among degrees. (Questionnaire 2) The interclass reliability of valence and arousal scores of SAM scale was calculated in this experiment. Cronbach’s α for valence scores was 0.837, arousal was 0.834.

### 2.5. Procedure

**Figure 2:**
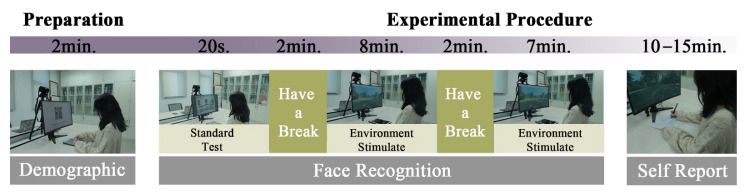
Preparation and experimental procedure.

**Figure A3:**
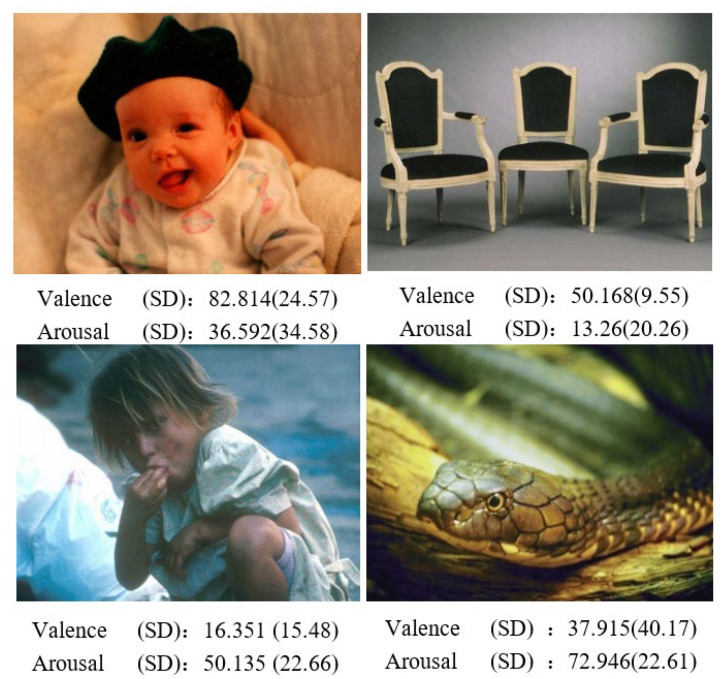
4 pictures randomly selected from GAPED 730 pictures.

Before the experiment, the participants were briefly introduced to the procedure. Then, they were asked to fill out a simple demographic questionnaire. Next, the participants were asked to view images from the Geneva Affective Picture Database (GAPED) [50]. The database has a wide selection of pictures with clear meanings and can convey emotional information relatively quickly. Four specific pictures, including positive, negative and neutral contents, were randomly selected from the database in order to proportionally cover the whole dimensional affective space (Figure A3). The evaluation results formed the benchmark for evaluating its impact. The pictures were displayed on PPT, and each picture was played for 5 s. After that, each participant was asked to watch 15 scenic videos in a random order. After 8 scenes, they rested for a period to avoid visual fatigue. The participants were asked to imagine what feelings they experienced in the scene and what their emotions were like in this place, rather than focusing on the scene depicted based on the video itself. After the experiment, the participants were asked to complete a questionnaire, including a SAM scale, and to evaluate the perceived plant attributes (Table A2) and the experience of the scene. (Figure 2).

### 2.6. Statistical Analysis

The collected data were arranged in Excel and transferred into IBM SPSS 21.0 (IBM Corp., Armonk, NY, USA). Our analyses focused on estimating the effect of spatial quality on emotional responses to UGS. The spatial quality values of each scene and emotional scores were then calculated. Pearson correlation analysis was used to show the relationships between spatial quality and emotion scores. We then entered the correlated spatial quality attributes in linear regression analysis with valence or arousal measured in different ways as the dependent variables. We examined the significant coefficients for the relationships between the attributes and predictive likelihood of valence and arousal. Analysis of variance and post hoc analysis were employed to examine the differences in valence or arousal for the UGS with various spatial structural attributes. Finally, an independent sample T test was conducted to present the differences in the participants’ sociodemographic characteristics.

## 3. Results

This section may be divided by subheadings. It should provide a concise and precise description of the experimental results, their interpretation, as well as the experimental conclusions that can be drawn.

### 3.1. Spatial Quality Related to Emotional Responses for UGSs

The descriptive statistics shown in Table 2 reveal that the values of spatial quality variables differed substantially across the plots in our samples. Pearson’s rho correlation analysis describes the relationship between the emotional responses and the variables of the spatial quality for UGSs. Based on these results, only B1 and D2 were not related to valence and arousal measured by SAM scales. For the face recognition method, A1, A2, A4, A5, B1, B2, B3, and D2 were associated with Vfr. A4, D1, and D2 were associated with the Afr.

### 3.2. Effects of the Interaction of UGS Spatial Quality on Emotional Responses

Linear regression analysis was conducted using backward regression analysis. This composite method was applied to determine whether there were several regression models involving different variable combinations that had significant relations with people’s emotions. To verify the model, we performed an analysis of variance and evaluated multicollinearity.

Table 3 shows 4 models from backward regression inclusions. The research focused on the model with high R^2^. The spatial quality of UGS accounted for 49.7% of the explained variance in the likelihood of Vsam. The most predictive variables, as indicated by the b-values, were C1 and C2. The results showed that A1, A2, A3, A5, B3, C1, C2, and D1 positively predicted the self-reported valence of UGS. In contrast, A4 was shown to be a negative predictor of Vsam. When related indicators were entered in the regression analysis model, they accounted for 46.1% of the explained variance in the likelihood of Asam. In summary, A1, A2, A3, A4, A5, B2, C1, and D2 were proven to be predictors of Asam. Among them, A1, A2, A3, A5, and D2 had a positive effect on the Asam model. Interestingly, A4 also showed a negative effect on Asam. The Vfr and Afr models showed weak predictive ability. In general, each of the 12 indicators of UGS, regardless of their contribution to the model, was of vital significance, as the model was meant to assess the predictive ability of all variables.

### 3.3. Effects of the Spatial Structure Attributes of UGSs on Emotional Responses

**Figure 3:**
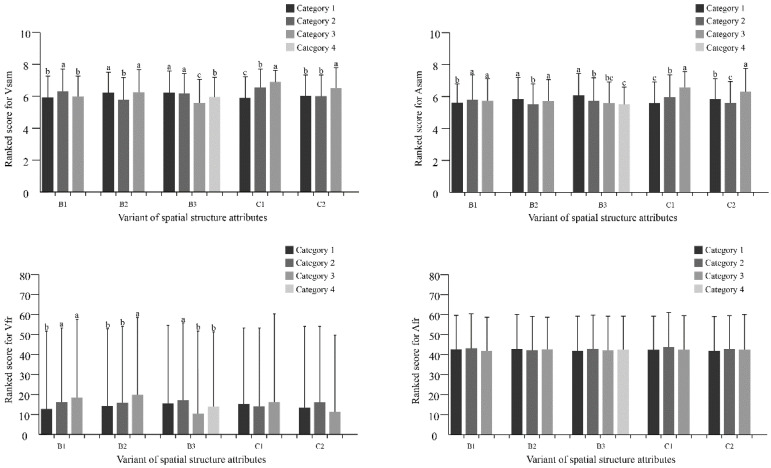
Post hoc test of the spatial structure attributes; Significant difference at the 0.05 level is shown by different letters (a, b and c).

Our research ranked the quantitatively measured spatial structure attributes according to established standards in Table 1 to perform ANOVA and post hoc analysis. We performed the homogeneity test of variance at the very beginning. If the data conformed to a normal distribution, the LSD method was used for pairwise comparison. Otherwise, we chose the Tamhane method for comparison, as shown in Figure 3 [51].

**Figure 4:**
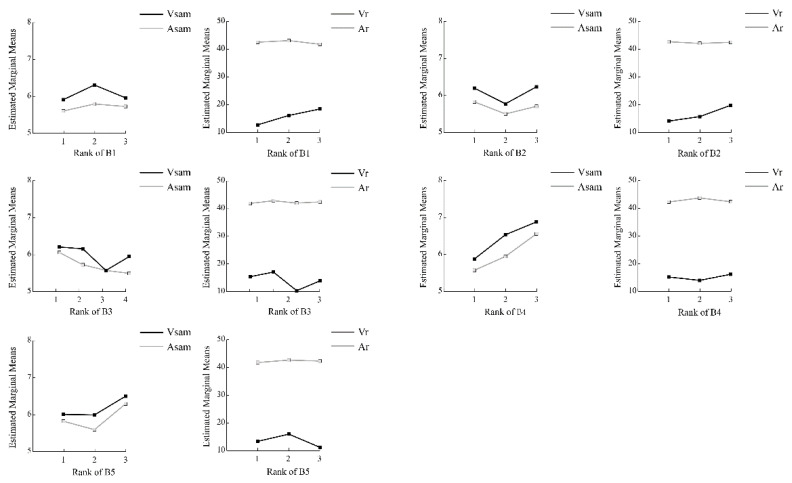
The extent of emotional responses differences of spatial structure attributes across the categories of scenes. Note: See Table 1 for the full abbreviations.

All related structural factors showed significant differences in Vsam and Asam (Figure 3). The significant heterogeneity of B1 can be observed for emotional responses. As shown in Figure 4, the 0.3-0.8ha^2^ were rated significantly higher than others in Vsam (F = 40.889, *p* = 0.000) and Asam (F = 9.297, *p* = 0.000). And the Vr increased with increasing of the B1(F = 11.652, *p* = 0.000). In addition, significant differences in Vsam (F = 63.820, *p* = 0.000) and Asam (F = 34.578, *p* = 0.000) were found between B2 levels, and the value of B2 from 1.5 to 2.5 conditions was rated significantly lower than others. For B3, Vsam decreased with increasing density (between 20–80%) and then increased slightly at 80%-100% (Figure 4). The Asam of B3 showed a trend of negative correlation as a whole. The main effect of C1 on both Vsam (F = 150.312, *p* = 0.000) and Asam (F = 100.988, *p* = 0.000) showed strong consistency. A higher percentage of water often leads to a higher valence and arousal. C2 showed similar conclusions, and the percentage of 80–100% was significantly higher than the others on Vsam and Asam. The face recognition results of ANOVA indicated that the main effects of B1, B2 and B3 on Vfr were significantly different (Figure 3). Post hoc analysis of B1 showed that the comparison result of the average scores of the groups with more obvious differences is “3 > 2 > 1” (F = 11.652, *p* = 0.000). Similarly, the Vfr on the 3rd rank of B2 was significantly higher than the 1st and 2nd rank (F = 4.269, *p* = 0.014). Moreover, B3 at 40–60% was considered the most pleasing (F = 6.865, *p* = 0.000). However, the results of ANOVA indicated that the main effects of spatial structure attributes on Afr were nonsignificant.

### 3.4. Gender Difference

Independent sample T tests were primarily conducted to show the differences in the participants’ sociodemographic characteristics (Table 4). The P value of the T test for gender was less than 0.01 under the conditions of the two methods, indicating that the valence and arousal values significantly differed by gender. For the V value, the results of Vsam and Vfr both showed higher values for women than men. For the A value, the results of Asam showed that women’s arousal was higher than that of men; nevertheless, Afr results showed that men’s arousal was higher than that of women.

## 4. Discussion

### 4.1. Driving Influence of the Spatial Quality Attributes of UGSs on Emotional Responses

The present study identified and described the influence of the spatial quality of UGSs based on emotional assessment dimensions. In this study, we used objective measures of spatial structure attributes, subjective measures of perceived plant attributes, and the experience of UGS to predict people’s emotional responses. The results showed relevant spatial quality that could significantly influence emotions. There are similarities between our findings and earlier research results, and some of these similarities are described above.

We combined spatial quality attributes to predict valence and arousal. The most predictive variable of the likelihood of Vsam was C2, followed by C1. Moreover, C1 was the most predictive attribute of Asam. This result replicates previous findings that trees and water easily draw individuals’ attention. Scenes with a higher percentage of trees and water are conducive to satisfying our biological needs and evoking positive emotional responses [43]. The contributions of D1 and D2 to the regression models were also confirmed. Thus, it is important to consider the activities and goals that drive individuals to visit green spaces [26,52]. In agreement with our expectations, individuals who tend to visit green spaces more frequently are more capable of perceiving their emotional well-being and, consequently, of receiving greater benefits from the green space experience. Note that A4 had a negative effect. This finding is supported by recent research showing that greenness can improve emotional well-being [53]. In other words, variegated colors may seem confused and have negative effects on emotion. The R^2^ values of Vfr and Afr were low, meaning that apart from the studied attributes, other attributes that were not included in the current study may also affect emotional responses. Our study aims to evaluate the predictive power of each variable, regardless of the degree to which the attribute explains the model.

The design of our study as well as the research questions we attempted to investigate only allow us to sketch the assessment framework for a specific type of UGS spatial quality that possibly contribute to emotional responses, and the environmental indicators may differ in various types of places [54]. We do not try to define a complete set of indicators in this research, and future research is needed to explore a complete series of spatial variables related, to the greatest extent possible, to specific places. The results should not be seen as a limitation of the design process but rather as a guide to understanding some of the spatial qualities of emotional well-being derived from UGS. It will be interesting and important to reconsider which spatial quality contributes to the enhancement of the valence and arousal of UGSs.

### 4.2. The Relationship between Spatial Structural Attributes and Emotional Responses

With regard to the quality of single attributes, differences were found for every scene. This differentiation indicated that we could perform a multidimensional emotion assessment of UGS, with respect to the given variability of the condition of structural attributes measured quantitatively [13].

The issue of size, usually synonymous with scale, is commonly discussed in landscape architecture and city planning theory. In general, our results support the claim that the higher value of valence was found in the medium to large UGS size. That is, the larger a green space, the more likely it is that a person will promote his/her positive emotion there. However, oversize areas provide more natural space for people, and they may appreciate the tranquility and reflection there, which tended to cause a more inactive emotion [18,55].

The results regarding the shape index of edges were mixed. It is commonly believed that the larger the shape index values are, the richer the spatial hierarchy [24]. Our results showed that the highest Vfr score was found in the scenes representing a scene with a high level of shape complexity. For Vsam and Asam, the lowest and the highest levels of shape complexity caused higher emotion scores compared with the middle level. A possible explanation is that the change in the shape index inevitably affects other structural attributes and spatial configurations, which in turn affects people’s emotions.

Canopy density showed significant differences in the results of the Vfr, Vsam, and Asam models. In terms of Vfr and Vsam, the low- and medium-density canopies had the highest scores. Regarding Asam, the lower the canopy density was, the higher the arousal. Canopy density had a greater impact on the enclosure of green spaces. Kaplan et al. (1989) believed that moderately open scenes were highly preferred [2]. Bjerke et al. (2006) proposed that people were more inclined to open grasses dotted with groups of trees and shrubs [56]. The results may provide some enlightenment to later research; that is, too high a density may exert a negative impact on people’s valence [57].

ANOVA and post hoc analysis investigated the independent contribution of water to emotional response. This research showed that a large percentage of water was rated more positively than scenes with less water in both Vsam and Asam. Overall, this study suggested a positive relationship between water area and emotional responses. The significant contributions of the percentage of water to the spatial quality of UGSs have been mentioned in numerous previous studies. These findings were also confirmed by the abovementioned results of our study. This importance may be because certain visual properties of aquatic environments have been proven to be attractive [43], and the experiences of aquatic environments were thus good for human mental well-being [29,58,59].

The percentage of plants in different scenes constitutes another point of concern. The results presented here suggest that higher tree density means higher Vsam. To ensure a moderate value of Asam, the percentage of plants should be no less than 20%. The same results were found in research by Kang et al. (2019): places where green vegetation is denser seem to exert positive impacts on the degree of human happiness [54]. In addition, season may influence participants’ emotional responses [60]. The pictures were taken in midsummer, and the laboratory experiments were performed at the same time. A higher percentage of plants might be more desirable in the summer because of tree shading or cooling effects. Future studies should explore emotional responses in other seasons.

### 4.3. Demographic Characteristics

Our research analyzed whether demographic differences played an important role in emotional responses. The results indicated that women experienced a significantly higher valence than men. Thus, the findings suggest that female participants were able to obtain significant valence from exposure to the environment stimuli experience, but male visitors’ expressions were not very sensitive. Our results concur with those in a recent study, which showed that male visitors generally showed neutral emotions in forest environments, while female visitors showed a positive contribution of such scenes to expressional emotions [16]. The results of arousal showed an inconsistent trend. Both biological and social differences between men and women might explain gender differences in emotional responses [17]. We hope that this kind of research will enable planners to focus on individual differences in the public and make urban planning more sophisticated and humane. The current understanding of the emotional responses and demographics of the UGS experience is still limited. More studies are needed to detect the mechanism for the variation of emotional responses in UGSs.

### 4.4. The Differences between the 2 Emotional Measurement Methods

According to these results, the physiological assessments of emotions do not exactly match the psychological assessments, even for the same attributes. One could ask what different attributes mean with regard to the valence and arousal measured by the two measures. The present results indicate their distinctiveness in that, as described above, each dependent variable has a somewhat different set of indicators, and each indicator mediates relations between emotional responses and UGSs somewhat differently. On the one hand, self-reporting emotions and objectively measured emotions are essentially different emotional attributes. On the other hand, we can interpret such differences as an inability of objective assessment to recognize all subjective characteristics of the emotions expressed on faces [40]. However, the method of self-reporting is simple. As Ulrich (1983) pointed out, the passive intellectual contemplation of a natural setting can be quite beneficial in itself, and therefore, post cognitive emotional responses may themselves be considered a reliable measure [1]. The method of face recognition is more objective, as it allows for real-time and continuous measurement and has been already used in psychological and behavioral studies, and its validity is well established [17,35,55]. Barrett et al. (2019) concluded that even more important than technology development, it is important for scientists to consider emotions in a more complex fashion [61]. In other words, the findings provide professionals with evidence regarding the quantitative indicators to reach moderate levels of emotional response. Nevertheless, the indicator set only contains initial theoretical foundations for moving towards multidimensional, specific and holistic planning guidelines for UGSs.

### 4.5. Limitations and Future Perspectives

Our approach also has some limitations. First, we tried to capture emotional responses in an experimental setting using simulated environments. Previous studies have shown that the influence of the environment on emotional responses acts on multisensory stimulation, such as visual, auditory, tactile, and olfactory stimulation [51]. The perception of green space among participants may be limited. However, an actual visit to the UGS may not always provide an ‘objective’ or more valid representation, as the physical characteristics of environments depending on the weather, time of the day, season, contingencies and so on, which might dramatically affect the participants’ judgments [56]. Self-evidently, such problems are eliminated when 360° visual simulation techniques are used, and an immersive experience of the environment is presented. Thus, we think that the use of a visual simulation method appropriately matched the objectives of our study. Second, as already mentioned, the results might be affected by the diversity of faces in the trained data sets. Therefore, we used the GAPED as the preintervention measurements of mood to standardize the dataset. In addition, people may not always express emotions explicitly through either facial expressions or text [35,62]. Barrret et al. (2019) found that people do many other things with their faces regardless of whether they are happy or sad [61]. For example, a smile can be mocking or ironic. Further exploration should be conducted to show the connections between human emotions and facial expressions on technology-mediated platforms [54]. A further limitation concerns the size and characteristics of the sample. As the emotional responses of UGSs depend on age, education background, socioeconomic status or other factors, a deep insight into individual differences in evaluating spatial quality of UGSs could merit attention in future research. Last but not least, we recognized that we treated greenspace as a single type of object instead of breaking it down by categories of type. Whilst we advocate the need to not treat greenspace as homogenous spaces, for practicality reasons that green spaces in the West Lake had a significant weight in the total amount of green spaces in Hangzhou, China. Exploring more comprehensive types of urban green spaces and exploring the impact of green space quality on individuals’ emotions is an important aspect for further research [7].

## 5. Conclusions

With the growing emphasis on the well-designed urban environment, the design of green space to enhance the mental health of people living in cities is of considerable importance. This study is an initial effort to identify the influence of the spatial quality for UGSs on people’s emotional responses. We revealed that a series of spatial qualities, including perceived plant attributes, spatial structure attributes and experiences of UGS, can elicit a range of significantly different valence-arousal emotion values of people. It became evident that no structural attribute is valuable or superfluous per se but that every attribute can be adjusted to promote people’s emotional well-being. 

Our findings can help designers make evidence-based decisions about the spatial quality of green spaces that will contribute to the health and wellbeing of the individuals. According to our research, when the size was no less than 0.3 ha^2^, the positive emotion was enhanced. Canopy density was negatively correlated with the emotional responses, and the valence and arousal were most significant when the value was 40–60%. This shows that the overall crown threshold for the planting of tall trees in UGSs should not be too dense. The emotion value increased as the percentage of plants and water increased, so designers are encouraged to incorporate plant and water elements into UGSs. The shape index of edges was interrelated with the other attributes, designers were encouraged to take them all into account while creating a high-quality UGS.

In terms of comparison between physiological and psychological methods, we found that the face recognition method was capable of assessing participants’ emotional responses to UGSs. The method has achieved objective results in our experiment. There are similarities between our findings and previous research results, indicating that face recognition is effective to some extent. Both physiological and psychological methods are useful, and we recommend combining them in future studies.

The results presented here should be considered with a view to future urban planning, where UGSs can be seen as a resource of importance to public mental health. However, before spatial quality can be used by practitioners as tools to promote health through design and urban planning, more research is needed to investigate a broader set of spatial attributes and improve the accuracy and pertinence of the emotion assessment model in UGS.

## Figures and Tables

**Table 1:** Scale of spatial quality for sample plots.

Attributes	Variable	Description	Categories					
			1	2	3	4	5	6
Emotion Dimensions	Vsam	Valence measured by SAM scale						
	Asam	Arousal measured by SAM scale						
	Vfr	Valence measured by Face recognition						
	Afr	Arousal measured by Face recognition						
Perceived Plant Attributes	A1. Vegetation Layers	Whether the layers of vegetation are abundant						
	A2. Shape of Trees	Whether the shape of trees is beautiful						
	A3. Texture of Trees	Whether the tactile sense of trees is smooth						
	A4. Color Diversity	Whether the UGS is rich in color						
	A5. Culture Connotation	Whether the UGS is rich in culture atmosphere						
Spatial Structure Attributes	B1. Size	Area of urban green space	<0.300	0.300–0.800	>0.800			
	B2. Shape Index of Edges	The ratio of the actual length of the space boundary to the circumference of the same area	<1.500	1.500–2.500	>2.500			
	B3. Canopy density	The ratio of the total crown width of the tree to the total area of the urban green space	0.200–0.400	0.400–0.600	0.600–0.800	0.800–1.000		
	C1. Percentage of Water	The number of water pixels in each of the panoramic photo	0.000–0.100	0.100–0.200	0.200–0.300			
	C2. Percentage of Plants	The number of plant pixels in each of the panoramic photo	0.000–0.2000	0.200–0.400	0.400–0.600	0.600–0.800	0.800–1.000	
Experiences of Urban Green Space	D1. Visit Frequency	The frequency you visit the UGSs	Almost Everyday	2 or 3 Times a Week	2 or 3 Times a Month	2 or 3 Times a Quarter	2 or 3 Times a Year	Almost Never
	D2. Use Function	The factors shown in the video that motivate you to use	Soothing or Not	Beautiful or Not	Relaxing or Not	Passable or Not	Lively or Not	Stationary or Not

**Table 2:** Descriptive statistics and Pearson’s rho correlation analysis of the spatial quality attributes.

Variable	Min.	Max.	M	S.D.	Vsam	Asam	Vfr	Afr
A1	1	5	3.584	0.809	0.562 **	0.538 **	−0.057 **	0.013
A2	1	5	3.531	0.844	0.602 **	0.599 **	−0.049 **	0.012
A3	1	5	3.271	0.813	0.511 **	0.512 **	−0.023	0.022
A4	1	5	3.086	0.906	0.383 **	0.399 **	−0.094 **	−0.025 *
A5	1	5	3.006	0.882	0.440 **	0.423 **	−0.096 **	0.021
B1	0.024	1.465	0.577	0.000	0.013	0.018	0.060 **	−0.009
B2	0.610	2.900	1.543	0.585	−0.030 *	−0.082 **	0.047 **	0.010
B3	0.350	1.000	0.626	0.222	−0.038 **	−0.076 **	−0.032 *	0.006
C1	0.000	0.256	0.031	0.070	0.212 **	0.179 **	0.001	0.013
C2	0.455	0.806	0.668	0.090	0.136 **	0.078 **	0.002	0.004
D1	0	6	3.647	0.871	0.434 **	0.382 **	0.007	0.040 **
D2	0	6	2.347	1.428	−0.017	−0.011	0.121 **	0.155 **

Note. We report Pearson correlations. * *p* ≤ 0.05 (1-tailed). ** *p* ≤ 0.01 (2-tailed).

**Table 3:** Linear regression analysis of the spatial quality and emotional responses.

Dependent	Independent	Unstandardized Beta		t	Sig.	Collinearity Statistics	
		B	Standard Error			Tolerance	VIF
Vsam r^2^ = 0.497 (F = 670.019, *p* = 0.000)	A1	0.465	0.021	21.966	0.000	0.605	1.654
	A2	0.470	0.023	20.790	0.000	0.489	2.045
	A3	0.318	0.022	14.714	0.000	0.576	1.737
	A4	−0.133	0.020	−6.655	0.000	0.543	1.842
	A5	0.145	0.021	7.010	0.000	0.530	1.887
	B3	0.295	0.064	4.620	0.000	0.884	1.131
	C1	2.056	0.204	10.055	0.000	0.860	1.163
	C2	1.132	0.160	7.063	0.000	0.847	1.181
	D2	0.153	0.011	14.261	0.000	0.758	1.319
Asam r^2^ = 0.461 (F = 579.961, *p* = 0.000)	A1	0.438	0.022	20.015	0.000	0.618	1.618
	A2	0.524	0.024	22.203	0.000	0.488	2.049
	A3	0.352	0.023	15.601	0.000	0.574	1.742
	A4	−0.044	0.021	−2.101	0.036	0.542	1.845
	A5	0.090	0.022	4.142	0.000	0.527	1.896
	B2	−0.126	0.025	−5.114	0.000	0.925	1.082
	B3	−0.106	0.064	−1.666	0.096	0.964	1.038
	C1	0.720	0.211	3.406	0.001	0.876	1.141
	D2	0.083	0.011	7.433	0.000	0.757	1.320
Vfr r^2^ = 0.020 (F = 20.409, *p* = 0.000)	A4	−2.162	0.843	−3.823	0.010	0.595	1.681
	A5	−5.007	0.900	−5.703	0.000	0.550	1.817
	B1	3.239	1.231	4.192	0.009	0.995	1.005
	B2	2.757	1.014	3.649	0.007	0.986	1.014
	D1	3.680	0.679	9.062	0.000	0.994	1.006
Afr r^2^= 0.019 (F = 40.537, *p* = 0.000)	A4	−0.514	0.307	−1.674	0.094	0.855	1.170
	D1	3.093	0.297	10.411	0.000	0.986	1.014
	D2	0.430	0.195	2.207	0.027	0.853	1.172

Note: See Table 1 for the full abbreviations.

**Table 4:** Independent sample T tests of gender differences.

Dependent Gender		M	S.D.	Sig.
Vsam	Male	5.929	1.396	0.000 **
	Female	6.122	1.524	
Vfr	Male	12.499	38.627	0.000 **
	Female	17.455	39.858	
Asam	Male	5.579	1.403	0.000 **
	Female	5.785	1.539	
Afr	Male	44.319	17.169	0.000 **
	Female	40.834	17.790	

Note. We report ** *p* ≤ 0.01 (2-tailed).

## Data Availability

The data are not publicly available due to the ongoing research, and the authors will continue to work with it in the future.

## Appendix A

**Table A1:** Descriptive statistics for sample plots.

Plot Number	Vsam	Asam	Vfr	Afr	Vegetation Layers	Shape of Trees	Texture of Trees	Color Diversity	Culture Connotation	Size	Shape Index of Edges	Canopy Density	Percentage of Water	Percentage of Plant	Visit Frequency	Use Function
1	6.529	5.500	16.033	44.106	3.647	3.647	3.471	3.000	3.059	0.116	2.500	1.000	0.000	0.796	3.647	2.265
2	6.882	6.559	16.202	42.367	4.000	3.941	3.441	3.471	3.324	1.258	1.390	0.575	0.256	0.591	3.647	2.971
3	6.441	6.118	12.473	43.947	4.000	3.824	3.206	3.441	3.235	0.171	1.000	0.576	0.107	0.670	3.647	2.441
4	5.382	5.294	14.419	43.422	2.971	3.294	2.971	2.647	2.794	0.252	1.320	1.000	0.000	0.455	3.647	2.000
5	6.500	6.294	11.215	42.327	3.676	3.824	3.500	3.000	2.794	0.394	0.610	0.394	0.000	0.806	3.647	2.971
6	6.235	5.706	19.806	42.519	3.735	3.706	3.647	3.118	3.382	0.500	2.900	0.489	0.000	0.788	3.647	2.382
7	5.618	5.147	19.044	42.483	3.265	3.500	3.235	2.824	2.794	1.408	1.670	0.414	0.000	0.612	3.647	1.882
8	5.765	5.618	9.475	39.640	3.853	3.588	3.206	3.265	3.294	0.166	0.740	0.831	0.000	0.598	3.647	2.000
9	5.529	5.324	8.098	42.113	3.618	3.265	3.147	3.235	2.853	0.215	1.700	0.726	0.000	0.657	3.647	2.500
10	5.441	5.382	18.777	40.890	3.147	3.176	2.971	2.794	2.853	0.900	1.790	0.510	0.000	0.656	3.647	2.029
11	6.618	5.765	15.657	43.518	3.941	3.676	3.441	3.176	2.941	0.630	1.080	0.484	0.110	0.699	3.647	2.588
12	6.118	5.559	15.705	42.458	3.441	3.324	3.147	2.882	3.000	0.088	1.460	1.000	0.000	0.660	3.647	2.382
13	5.882	5.412	17.643	44.063	3.412	3.353	3.353	3.176	2.853	0.775	1.410	0.444	0.000	0.603	3.647	2.412
14	5.618	5.824	12.670	41.869	3.588	3.382	3.059	3.294	2.853	0.024	1.920	0.605	0.000	0.754	3.647	2.000
15	5.912	5.824	19.626	41.380	3.471	3.471	3.265	2.971	3.059	1.465	1.660	0.348	0.000	0.681	3.647	2.382

**Table A2:** Short version revised scale for perceived plant attributes.

Variable	Description	Scales				
		1	2	3	4	5
A1. Vegetation Layers	Whether the layers of vegetation are abundant					
A2. Shape of Trees	Whether the shape of trees is beautiful					
A3. Texture of Trees	Whether the texture of trees is smooth					
A4. Color Diversity	Whether the UGS is rich in color					
A5. Culture Connotation	Whether the UGS is rich in culture connotation					

**Questionnaire 1.** Short-version revised scale for experience of UGS

1. Do you think any factor shown in the video will motivate you to use the scene? (Multiple Choice)
  (1) Is the scene soothing? □Yes □No
  (2) Is the scene beautiful? □Yes □No
  (3) Is the scene relaxing? □Yes □No
  (4) Is the scene accessible? □Yes □No
  (5) Does the scene provide rich activities? □Yes □No
  (6) Does the scene provide a place to stay? □Yes □No
2. ‘How often do you visit the UGSs shown in the videos. (Exclusive Choice)
  □ almost every day
  □ 2 or 3 times a week
  □ 2 or 3 times a month
  □ 2 or 3 times a quarter
  □ 2 or 3 times a year
  □ almost never

**Questionnaire 2.** SAM scale

1. Please select the degree of pleasure you feel after watching the panoramic video of the scene. The numbers 1, 3, 5, 7 and 9 represent frown (a frowning figure), unhappy, neutral, smiling, and happy (a smiling figure), and 2, 4, 6, 8 are provided you with subtler choices among degrees.

2. Please select the degree of arousal you feel after watching the panoramic video of the scene. The numbers 1, 3, 5, 7 and 9 indicate sleepy (eyes closed), drowsy, neutral, excited, and stimulation (eyes wide open), and 2, 4, 6, 8 are provided you with subtler choices among degrees.

http://view.vryun.work/#/panoview/50227 [Online; accessed on 6 August 2021].

http://view.vryun.work/#/panoview/50226 [Online; accessed on 6 August 2021].
